# Kidnapping model: an extension of Selten's game

**DOI:** 10.1098/rsos.171484

**Published:** 2017-12-06

**Authors:** Azhar Iqbal, Virginie Masson, Derek Abbott

**Affiliations:** 1 School of Electrical & Electronic Engineering, University of Adelaide, South Australia 5005, Australia; 2 School of Economics, University of Adelaide, South Australia 5005, Australia

**Keywords:** game theory, kidnapping, Selten's model of kidnapping, subgame perfection

## Abstract

Selten's game is a kidnapping model where the probability of capturing the kidnapper is independent of whether the hostage has been released or executed. Most often, in view of the elevated sensitivities involved, authorities put greater effort and resources into capturing the kidnapper if the hostage has been executed, in contrast with the case when a ransom is paid to secure the hostage's release. In this paper, we study the *asymmetric game* when the probability of capturing the kidnapper depends on whether the hostage has been executed or not and find a new uniquely determined perfect equilibrium point in Selten's game.

## 1. Introduction

Although it is not a common crime, there are parts of the world where kidnapping is a real and constant threat. In a typical scenario, a victim is abducted and a monetary demand is made for his or her release. Although it appears to be a simple exchange of money for the release of a hostage, dealing with the situation does require considerable planning. This is where game theory [1–3], a developed branch of mathematics that models strategic situations, can offer valuable insights.

In 1976, Selten [4] developed a game-theoretic model of kidnapping as a two-person sequential game between player K (Kidnapper) and player F (hostage's Family). The game begins with K's choice whether or not to go ahead with his plan that is described by a binary decision variable *b*:

1.1 $$b:\left\{ \begin{array}{ll} 0 & \text{not to kidnap,}\\ 1 & \text{to kidnap.} \end{array}\right.$$

The game ends if K selects *b*=0. If K selects *b*=1, he takes the hostage to a hidden place unknown to player F and to the police, and announces a ransom demand *D*.

Numerous questions then arise. Will the hostage's family pay the ransom *D* or will they try to negotiate a lower amount? If they do pay the ransom, should K free the hostage instead of executing him/her? Moreover, if F does not expect K to free the hostage, why should it be expected that F pay some ransom?

It is assumed that, on knowing the demand *D*, a negotiation process starts between players K and F. Player F makes an offer *C* which is the amount willing to be paid, and player K either decides to accept *C* and release the hostage, or to execute the hostage. The situation can be seen as a simple description of an extended bargaining process.

In his model, Selten assumed that K's threat to execute the hostage has some credibility, even though K cannot improve his situation by doing so. In particular, it is expected that with a positive probability *α*, player K may deem the offer *C*<*D* to be insufficient, and thus decide to execute the hostage. Selten assumed that the probability *α* can be described as a linear function of *C*/*D*:

1.2 $$\alpha =a\left[1-\frac{C}{D}\right]\;\text{for}\;0\leq \;C\leq D,$$

where *a* is a constant with 0<*a*<1. The parameter *α* thus describes K's non-rational decision to execute the hostage, as in this case the traditional utility maximization principle is ignored.

It is nonetheless possible that player K makes the rational decision to execute the hostage independently of whether the offer *C* is deemed insufficient or not. Selten used a binary decision variable *e* to describe this situation:

1.3 $$e:\left\{ \begin{array}{ll} 0 & \text{release of hostage for ransom }C,\\ 1 & \text{execution of hostage,} \end{array}\right.$$

i.e. even if an offer is made at *C*, 0≤*C*≤*D*, the hostage can still be executed for some *C*.

## 2. Modified kidnapping game

In either case of the hostage having been executed or released, the authorities will put efforts in finding and capturing the kidnapper K. In Selten's model, it is assumed that, in both cases, the authorities will be successful in capturing K with some probability *q*, where

2.1 0<q<1,

i.e. the probability of capturing the kidnapper is independent of whether the hostage has been released or executed. We ask whether this indeed is the usual case.

Cursory observations of media coverage related to kidnapping incidences highlight the political pressure faced by authorities to severely punish those responsible, the idea being that punishment helps decrease the incidence of such events in the future. It is, however, unclear whether authorities favour the allocation of extra resources towards the capture of those who executed the hostage or those who did not. No consensus appears to prevail, and resource spending seems to be case dependent and government dependent, with the media likely playing a role in the decision of whether extra resources are spent to increase the chances of capturing the kidnapper.

Assuming that increased spending leads to a higher probability of capture, we thus adopt two random choice parameters *q*_0_ and *q*_1_ instead of the fixed probability *q* assumed by Selten, that we define as follows:

2.2 $$\begin{array}{rl} q_{0} & \;=\text{probability of capture of K if hostage is released}\\ \text{and}\;q_{1} & \;=\text{probability of capture of K if hostage is killed.} \end{array}\}$$

This allows us an improved modelling of the responses by authorities, and helps us elucidate whether allocating extra resources to increase the likelihood of capturing the kidnapper influences the kidnapper's strategy.

As we focus on the probability of the kidnapper's capture, it is also important to encompass the idea that, in the case where the hostage is executed, families derive a higher disutility from the kidnapper still being at large. This gives us the pay-offs depicted in table 1.

**Table 1.** The pay-offs for players K and F.

	pay-offs	
outcome	player K	player F
kidnapping does not take place	0	0
release of hostage for ransom payment *C*, kidnapper not caught	*C*	−*C*
kidnapper caught after release of hostage	−*X*	0
kidnapper not caught after execution of hostage	−*Y*	−*W*_1_
kidnapper caught after execution of hostage	−*Z*	−*W*_2_

Elevated sensitivities lead to increased pressure on the police, and government, to capture the kidnappers that usually results in an increase in the resources for finding the kidnappers. Investing extra efforts and resources may result in an increased probability of capturing the kidnapper.

In the following, we study the situation when the probability of capturing the kidnapper depends on whether the hostage has been killed or not and find a new uniquely determined perfect equilibrium point in Selten's game.

Here, *W*_1_, *W*_2_, *X*, *Y* and *Z* are positive constants and utilities of K and F that are assumed to be linear in money. In the original game presented by Selten, *W*_1_=*W*_2_=*W*, and *q*_0_=*q*_1_=*q*.

As in Selten's game, if K is caught, the execution of the hostage results in an increased disutility relative to the case when K releases the hostage. Thus,

2.3 Z≥X.

As the complete history of the previous game is known to both players at every point in the course of play, the game can be identified as an extensive game with perfect information [5].

**Figure 1.**
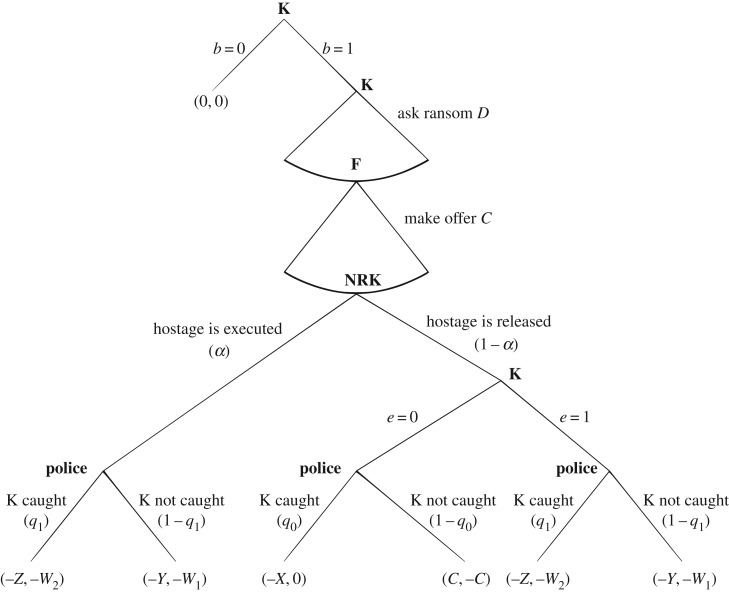
Representation of the game in extensive form. Strategic players, i.e. those deriving pay-offs from the game, are K (Kidnapper) and F (hostage's Family). NRK represents the non-rational decision of player K. NRK and the police's later actions represent random events occurring with some probabilities. Pay-offs are presented for player K first and player F second.

Note that table 1 encompasses a number of simplifying assumptions. First, player K's cost of preparing the kidnapping is assigned zero value. Also, player F's non-monetary disutilities, other than those that are incurred if player K executes the hostage, are ignored. In reality, there can be significant disutility for player F resulting from the emotional stress of engaging in a bargaining process with player K. Also, player F is assumed to gain no utility from the capture of K by the authorities if the hostage is released. Finally, note that utilities when player K is caught after the release of the hostage do not depend on the ransom money *C* as it is recovered and given back to player F. However, player K is then left with disutility *X*. Figure 1 shows the extensive form of the game.

### 2.1 Game timeline

Below, we provide a short description of the timeline of the game:

1. Player K chooses between *b*=1 and *b*=0, i.e. whether or not to kidnap someone.
2. If K selects *b*=0, the game ends and both players K and F receive zero pay-offs; if K selects *b*=1, K then announces demand *D*.
3. After observing demand *D*, F makes an offer *C* such that 0≤*C*≤*D*.
4. After K observes the offer *C*, a non-rational execution of the hostage occurs with probability *α* defined by equation (1.2).
5. If the hostage is not executed non-rationally, K chooses between *e*=0 and *e*=1. If K selects *e*=0, this means that ransom *C* is paid and the hostage is released. If K selects *e*=1, the hostage is (rationally) executed irrespective of whether any ransom is paid or not.
6. After release or execution of the hostage, two final random choice parameters *q*_0_ and *q*_1_ reflect the likelihood for the kidnapper to be captured, where *q*_0_ is the probability of capture if the hostage is released and *q*_1_ is the probability of capture if the hostage is executed.
7. The game then ends with pay-offs according to table 1.

## 3. Equilibrium of the game

Being one of the foundational concepts in game theory, Nash equilibria are used to predict the strategies used by players of non-cooperative games. A strategy profile specifies a strategy for each player and constitutes a Nash equilibrium if no player has an incentive to deviate from their current strategy. Any finite game admits at least one Nash equilibrium. The mathematical conditions defining a Nash equilibrium, called the Nash conditions, may nonetheless lead to unreasonable outcomes, as pointed out by Selten [6,5]. This is because the Nash conditions do not account for the dynamics of the game (if any).

Selten thus used the notion of *perfect equilibrium* as a refinement on the set of Nash equilibria to solve for the equilibrium of the Kidnapping game. A *subgame perfect equilibrium* [5–8] is not only a Nash equilibrium in the whole game, but it is also a Nash equilibrium in every subgame. For finite games with perfect information, such as the one considered in this paper, subgame perfect equilibria are commonly determined using backward induction [2,1].

In what follows, we follow Selten's original work, and identify the equilibrium of the game using the concept of subgame perfection [2,9,10].

### 3.1 Optimal choice of *e*

We start by examining the subgame that begins with player K's choice of *e*. Let *V*_0_ be K's expected pay-off if K selects *e*=0 and let *V*_1_ be the expected pay-off if K selects *e*=1 (i.e. the execution of the hostage). We have:

3.1 $$V_{0}=(1-q_{0})C-q_{0}X$$

and

3.2 $$V_{1}=-(1-q_{1})Y-q_{1}Z.$$

Note that the case

3.3 $$q_{0}=q_{1}=q$$

was considered by Selten.

In that case, as *C*≥0, *Y* >0, *Z*≥*X* and 0<*q*<1 we have

3.4 $$V_{0}>V_{1}$$

and *e*=0 becomes the optimal choice for player K. That is, for the case studied by Selten, player K will never rationally decide to execute the hostage. This could give the impression that when (3.3) does not hold, release of the hostage (*e*=0) would not remain the optimal choice of *e*. If the values of *q*_0_ and *q*_1_ rely on heightened sensitivities, i.e. authorities allocate more resources when the hostage has been executed so as to increase the likelihood of capturing K, we can assume that *q*_1_>*q*_0_. In this case, *e*=0 remains the optimal choice for K, as in Selten's original work. This is because (1−*q*_0_)*C*>0, while −(1−*q*_1_)*Y* <0, and thus disutilities are ranked such that *q*_0_*X*<*q*_1_*Z* (as *X*≤*Z*), which gives us that *V*_0_>*V*_1_.

However, if *q*_0_>*q*_1_, release of the hostage (*e*=0) may *not* remain the optimal choice for K, which is in contrast with Selten's work.

### 3.2 Optimal choice of *C*

In the subgame that begins with player F's choice of *C*, player F knows that player K can execute the hostage with probability *α* given in equation (1.2). Using table 1, the expected value of F's utility is thus equal to:

3.5 $$U=(1-\alpha )[(1-q_{0})(-C)+q_{0}(0)]+\alpha [(1-q_{1})(-W_{1})+q_{1}(-W_{2})].$$

With the constraints

3.6 $$W_{1}=W_{2}=W\;\text{and}\;q_{0}=q_{1}=q.$$

Equation (3.5) is reduced to

3.7 U=−(1−α)(1−q)C−αW,

which is player F's expected value of utility for the case that Selten considered. From figure 1, we note that (1−*α*) is the probability of the hostage being released because of K's non-rational decision. However, *e*=0 is K's rational decision to release the hostage and thus (1−*α*) is not the probability of *e*=0.

Using the expression for *α* from equation (1.2) in equation (3.5), we have:

3.8 $$\begin{array}{rl} U & \;=[-a(1-q_{0})]\frac{C^{2}}{D}+[(1-q_{1})W_{1}+q_{1}W_{2}]\frac{aC}{D}\\ & \;\;-(1-a)(1-q_{0})C-[(1-q_{1})W_{1}+q_{1}W_{2}]a. \end{array}$$

Once again, under the constraints described by (3.6) equation (3.8) reduces to

3.9 $$U=-a(1-q)\frac{C^{2}}{D}+\left[\frac{aW}{D}-(1-a)(1-q)\right]C-aW,$$

which is a strictly concave quadratic function as obtained by Selten [4]. To determine the optimal value $\bar{C}$ of *C* we compute ∂*U*/∂*C* from equation (3.8)

3.10 $$\frac{\partial U}{\partial C}=[-a(1-q_{0})]\frac{2C}{D}+[(1-q_{1})W_{1}+q_{1}W_{2}]\frac{a}{D}-(1-a)(1-q_{0}).$$

Equation (3.10) shows that *U* assumes its maximum at

3.11 $$C^{\prime }=\frac{(1-q_{1})W_{1}+q_{1}W_{2}}{2(1-q_{0})}-\frac{(1-a)D}{2a},$$

if the value of *C* is in the interval 0≤*C*≤*D*. This is the case if *D* is in the closed interval between the following critical values:

3.12 $$D_{1}^{\prime }=\frac{a}{\left(1+a\right)}.\frac{(1-q_{1})W_{1}+q_{1}W_{2}}{\left(1-q_{0}\right)}$$

and

3.13 $$D_{2}^{\prime }=\frac{a}{\left(1-a\right)}.\frac{(1-q_{1})W_{1}+q_{1}W_{2}}{\left(1-q_{0}\right)}.$$

As before, under the constraints (3.6) equations (3.12) and (3.13) become

3.14 $$D_{1}=\frac{a}{\left(1+a\right)}.\frac{W}{\left(1-q\right)}$$

and

3.15 $$D_{2}=\frac{a}{\left(1-a\right)}.\frac{W}{\left(1-q\right)}$$

as obtained by Selten. To determine the range for which *U* is an increasing function, i.e.

3.16 $$\frac{\partial U}{\partial C}>0,$$

we use equations (3.10) and (3.12) to write inequality (3.16) as

3.17 $$[(1+a)D_{1}^{\prime }-(1-a)D]>2aC,$$

i.e. the function *U*, as described by equation (3.8), is an increasing function for $D_{1}^{\prime }>D$. Likewise, considering the inequality

3.18 $$\frac{\partial U}{\partial C}<0,$$

we use equations (3.10) and (3.13) to write inequality (3.18) as

3.19 $$(D_{2}^{\prime }-D)(1-a)<2aC,$$

i.e. the function *U*, as described by equation (3.8), is a decreasing function for $D_{2}^{\prime }<D$.

In view of equation (3.11) describing the maximum that the function *U* assumes, player F's optimal offer ${\bar{C}}^{\prime }$ can be described as follows:

3.20 $${\bar{C}}^{\prime }=\left\{ \begin{array}{ll} D & \text{for}\;0<D\leq D_{1}^{\prime },\\ \frac{(1-q_{1})W_{1}+q_{1}W_{2}}{2(1-q_{0})}-\frac{(1-a)D}{2a} & \text{for}\;D_{1}^{\prime }<D\leq D_{2}^{\prime },\\ 0 & \text{for}\;D>D_{2}^{\prime }. \end{array}\right.$$

As *D* increases, the optimal offer ${\bar{C}}^{\prime }$ first increases up to $D_{1}^{\prime }$ and then decreases until it becomes 0 at $D=D_{2}^{\prime }$. In the interval $D_{1}^{\prime }<D\leq D_{2}^{\prime }$, the optimal offer ${\bar{C}}^{\prime }$ is decreased by an increase of *D*. The threat of execution of the hostage is avoided in the intervals $0<D\leq D_{1}^{\prime }$, as player F agrees to meeting the demand for the ransom.

Note that under constraints (3.6), the optimal offer ${\bar{C}}^{\prime }$ is reduced to $\bar{C}$

**Figure 2.**
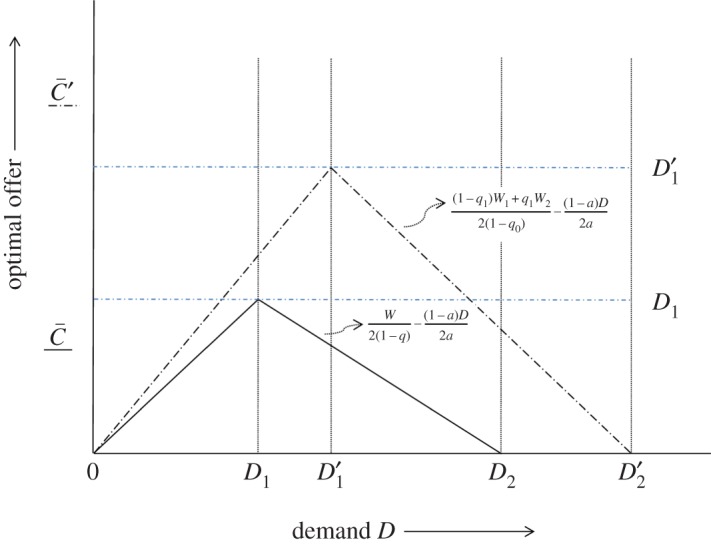
Optimal offer against the demand when the probability of capturing the kidnapper depends on whether the hostage has been executed or not (dotted line) and in the case studied by Selten (solid line). The scales along the optimal offer and demand axes are different.

3.21 $$\bar{C}=\left\{ \begin{array}{ll} D & \text{for}\;0<D\leq D_{1},\\ \frac{W}{2(1-q)}-\frac{(1-a)D}{2a} & \text{for}\;D_{1}<D\leq D_{2},\\ 0 & \text{for}\;D>D_{2,} \end{array}\right.$$

where *D*_1_ and *D*_2_ are given in equations (3.14) and (3.15), as obtained by Selten. Figure 2 plots the optimal offer against the demand *D* when the probability of capturing the kidnapper depends on whether the hostage has been executed or not (dotted line) and in the case studied by Selten (solid line). Note that, with reference to equations (3.14), (3.15), (3.12) and (3.13), the figure assumes that *D*_2_>*D*_1_ and $D_{2}^{\prime }>D_{1}^{\prime }$, but generally $D_{1}^{\prime }-D_{1}\neq D_{2}^{\prime }-D_{2}$ and $D_{2}-D_{1}\neq D_{2}^{\prime }-D_{1}$.

### 3.3 Optimal choice of *D*

We now consider the subgame that begins with player K's choice of *D*, the amount requested for the ransom. The optimal offer is given by equation (3.20). Let $\bar{\alpha }$ and ${\bar{V}}_{0}$ be the values that *α* and *V*_0_ assume at $C=\bar{C}$, respectively. Then, the optimal probability of the non-rational execution of the hostage as a function of demand *D* becomes:

3.22 $${\bar{\alpha }}^{\prime }=a\;\left(1-\frac{{\bar{C}}^{\prime }}{D}\right).$$

Using equation (3.1), we have

$${\bar{V}}_{0}=(1-q_{0}){\bar{C}}^{\prime }-q_{0}X.$$

Therefore, player K's expected pay-off becomes

3.23 $$V=(1-{\bar{\alpha }}^{\prime }){\bar{V}}_{0}+{\bar{\alpha }}^{\prime }V_{1},$$

where

3.24 $${\bar{V}}_{0}=(1-q_{0}){\bar{C}}^{\prime }-q_{0}X$$

and *V*_1_ is given by equation (3.2). Therefore,

3.25 $$V=a\;\left(1-\frac{{\bar{C}}^{\prime }}{D}\right)\;(V_{1}-{\bar{V}}_{0})+{\bar{V}}_{0}.$$

Using equation (3.20), *V* becomes:

3.26 $$V=\left\{ \begin{array}{ll} {\bar{V}}_{0} & \text{for}\;0<D\leq D_{1}^{\prime },\\ a\;\left[1-\frac{(1-q_{1})W_{1}+q_{1}W_{2}}{2(1-q_{0})D}+\frac{\left(1-a\right)}{2a}\right](V_{1}-{\bar{V}}_{0})+{\bar{V}}_{0} & \text{for}\;D_{1}^{\prime }<D\leq D_{2}^{\prime },\\ a(V_{1}-{\bar{V}}_{0})+{\bar{V}}_{0} & \text{for}\;D>D_{2}^{\prime }, \end{array}\right.$$

which can also be re-written as

3.27 $$V=\left\{ \begin{array}{ll} (1-q_{0})D-q_{0}X & \text{for}\;0<D\leq D_{1}^{\prime },\\ \frac{1}{2}\;\left[(1+a)-\frac{a[(1-q_{1})W_{1}+q_{1}W_{2}]}{(1-q_{0})D}\right]V_{1} & \\ \;+\;\left[\frac{\left(1-a\right)}{2}+\frac{a[(1-q_{1})W_{1}+q_{1}W_{2}]}{(1-q_{0})D}\right]{\bar{V}}_{0} & \text{for}\;D_{1}^{\prime }<D\leq D_{2}^{\prime },\\ a[(1-q_{1})(-Y)+q_{1}(-Z)]+(1-a)(-q_{0}X) & \text{for}\;D>D_{2}^{\prime }. \end{array}\right.$$

Consider first $D_{1}^{\prime }<D\leq D_{2}^{\prime }$. From equation (3.27), note that *V* is a decreasing function of *D* if ${\bar{V}}_{0}$ is a constant. But ${\bar{V}}_{0}=(1-q_{0}){\bar{C}}^{\prime }-q_{0}X$ and for $D_{1}^{\prime }<D\leq D_{2}^{\prime }$ we have ${\bar{C}}^{\prime }=((1-q_{1})W_{1}+q_{1}W_{2})/2(1-q_{0})-(1-a)D/2a$. An increase of *D* decreases ${\bar{C}}^{\prime }$ and thus ${\bar{V}}_{0}$ is decreased too. That is, *V* will be decreased further (relative to the case when ${\bar{V}}_{0}$ is assumed constant) when *D* increases within the interval $D_{1}^{\prime }<D\leq D_{2}^{\prime }$. So that *V* as a function of *D* first increases for values of *D* up to $D_{1}^{\prime }$. It then decreases for values of *D* up to $D_{2}^{\prime }$ and then remains constant. Recall that $D_{1}^{\prime }$ and $D_{2}^{\prime }$ are given in equations (3.12) and (3.13).

Note that under the constraints (3.6), equation (3.27) is reduced to

3.28 $$V=\left\{ \begin{array}{ll} (1-q)D-qX & \text{for}\;0<D\leq D_{1},\\ \frac{1}{2}\;\left[(1+a)-\frac{aW}{(1-q)D}\right]V_{1}+\left[\frac{\left(1-a\right)}{2}+\frac{aW}{(1-q)D}\right]{\bar{V}}_{0} & \text{for}\;D_{1}<D\leq D_{2},\\ a\;\left[(1-q)(-Y)+q(-Z)\right]+(1-a)(-qX) & \text{for}\;D>D_{2}, \end{array}\right.$$

as discussed by Selten, which is an increasing function for $0<D\leq D_{1}^{\prime }$. For $D_{1}^{\prime }<D\leq D_{2}^{\prime }$ in (3.28), as is the case for the function (3.27), if ${\bar{V}}_{0}$ is a constant then *V* is a decreasing function of *D*. But now ${\bar{V}}_{0}=(1-q)\bar{C}-qX$ as ${\bar{C}}^{\prime }$ reduces to $\bar{C}$, given by (3.21), under constraints (3.6). An increase of *D* decreases $\bar{C}$ and thus ${\bar{V}}_{0}$ is decreased too. That is, *V* will be decreased further (relative to the case when ${\bar{V}}_{0}$ is assumed constant) when *D* increases within the interval $D_{1}^{\prime }<D\leq D_{2}^{\prime }$.

Player K's optimal demand ${\bar{D}}^{\prime }$ can be considered as the highest demand $D_{1}^{\prime }$ such that player F's optimal offer ${\bar{C}}^{\prime }$ coincides with the demand. To determine ${\bar{D}}^{\prime }$ we refer to equation (3.20) and set

3.29 $${\bar{C}}^{\prime }={\bar{D}}^{\prime }\;\text{and}\;D={\bar{D}}^{\prime },$$

to have

3.30 $${\bar{D}}^{\prime }=\frac{(1-q_{1})W_{1}+q_{1}W_{2}}{2(1-q_{0})}-\frac{(1-a){\bar{D}}^{\prime }}{2a}$$

which gives

3.31 $${\bar{D}}^{\prime }=\frac{a}{\left(1+a\right)}.\frac{(1-q_{1})W_{1}+q_{1}W_{2}}{\left(1-q_{0}\right)}.$$

Equation (3.31), under the constraints (3.6), then gives the player K's optimal demand $\bar{D}$ as

3.32 $$\bar{D}=\frac{a}{\left(1+a\right)}.\frac{W}{\left(1-q\right)}$$

as obtained by Selten. Equation (3.32) shows that a higher value of *q* results in an increase in $\bar{D}$. This also shifts *D*_1_ and *D*_2_, given by equations (3.14) and (3.15), to higher values.

Thus, if the allocation of more resources to K's capture is linked to an increase in *q* then this also results in an increase in the optimal demand $\bar{D}$. Increasing *q* by allocating higher resources to police, however, is not as an effective policy as it appears to be. This is because with K's increased probability of capture, F's chances to get the ransom money back are also increased. This results in an increase in F's willingness to pay and thus to a higher optimal demand.

In our model, if the probability of capture *q*_1_ is increased, it also results in an increase in the optimal demand $\bar{D}$. However, since this increase only concerns *q*_1_, the likelihood of K to be captured once he executes the hostage, it does not have the perverted effect of increasing F's willingness to pay.

### 3.4 Optimal choice of *b*

The binary decision variable *b* in (1.1) describes player K's choice whether or not to go ahead with the plan to kidnap. The game ends if K selects *b*=0 and the hostage is kidnapped if K selects *b*=1. Considering the subgame which begins with player K's choice of *D*, the player K's pay-off expectation *V* is given by equation (3.27). As noted above, *V* as a function of *D* is first increasing up to $D_{1}^{\prime }$ and then decreasing up to $D_{2}^{\prime }$ and then remaining constant. The optimal value ${\bar{D}}^{\prime }$ of *D* is given by equation (3.31). As noted before equation (3.29), ${\bar{D}}^{\prime }$ is the highest demand $D_{1}^{\prime }$ such that player F's optimal offer ${\bar{C}}^{\prime }$ coincides with the demand. Let ${\bar{V}}^{\prime }$ be the value of *V* assumed at ${\bar{D}}^{\prime }$. From equation (3.27) we have

3.33 $$V=(1-q_{0})D-q_{0}X\;\text{for}\;0<D\leq D_{1}^{\prime },$$

then

3.34 $${\bar{V}}^{\prime }=(1-q_{0}){\bar{D}}^{\prime }-q_{0}X$$

and using equation (3.31) this can be written as

3.35 $${\bar{V}}^{\prime }=\frac{a[(1-q_{1})W_{1}+q_{1}W_{2}]}{\left(1+a\right)}-q_{0}X,$$

which at *q*_1_=*q*_0_=*q* becomes $\bar{V}=(a/\left(1+a\right))W-qX$ as in Selten's original work. This shows that if the probability of capture *q* can be increased by allocating additional resources to the efforts in finding K then the possibility of decreasing $\bar{V}$ is only limited by the availability of the resources. In our model, the increase of either probabilities, *q*_1_ or *q*_0_, leads to an overall decrease in K's utility, and the effect very much depends on the relative values of *X*, *W*_1_ and *W*_2_. In particular, an increase in *q*_1_ results in the optimal choice of *b* likely to be *b*=0, as the value identified in equation (3.37) decreases (keeping *q*_0_ constant), and thus the first condition is more likely to be satisfied. Similarly, if *q*_0_ increases (keeping *q*_1_ constant) then the first condition, i.e. *b*=0 is more likely to hold. If Δ*W*=*W*_1_−*W*_2_ is sufficiently large however, then increasing *q*_1_ appears to be more optimal in discouraging to select *b*=1.

Now the optimal choice of $\bar{b}$ is obtained by the following requirements:

3.36 $$\begin{array}{rl} {\bar{b}}^{\prime } & \;=0\;\text{for}\;{\bar{V}}^{\prime }<0\\ \text{and}\;{\bar{b}}^{\prime } & \;=1\;\text{for}\;{\bar{V}}^{\prime }>0, \end{array}$$

which can be written as

3.37 $${\bar{b}}^{\prime }:\left\{ \begin{array}{ll} 0 & \text{for}\;\frac{a[(1-q_{1})W_{1}+q_{1}W_{2}]}{\left(1+a\right)}<q_{0}X,\\ 1 & \text{for}\;\frac{a[(1-q_{1})W_{1}+q_{1}W_{2}]}{\left(1+a\right)}>q_{0}X \end{array}\right.$$

and when *W*_1_=*W*_2_=*W*, and *q*_0_=*q*_1_=*q*, it is reduced to

3.38 $$\bar{b}:\left\{ \begin{array}{ll} 0 & \text{for}\;\frac{aW}{\left(1+a\right)}<qX,\\ 1 & \text{for}\;\frac{aW}{\left(1+a\right)}>qX \end{array}\right.$$

as obtained by Selten. Player K's choice whether or not to go ahead with the plan to kidnap now depends on *W*_1_, *W*_2_, *q*_0_ and *q*_1_.

## 4. Discussion

The dependence of K's probability of being captured on whether he has executed the hostage or not can be represented as a bifurcation of *q* (probability of K's capture in either case of hostage having been executed or not) into *q*_1_ (probability of K's capture when the hostage has been executed) and *q*_0_ (probability of K's capture when the hostage has been released after paying the ransom).

Overall, we show that increasing either *q*_0_ or *q*_1_ leads to a reduced likelihood of kidnapping, provided that *q*_1_>*q*_0_ (otherwise *e*=0 is not necessarily the optimal choice of K). We also show that if the kidnapping took place, releasing the hostage and paying the ransom remains the optimal choice for K provided the motivation in assigning values to *q*_0_ and *q*_1_ takes into account the heightened sensitivities, i.e. authorities spend more resources when the hostage has been executed so as to increase the likelihood of capturing player K (i.e. *q*_1_>*q*_0_). Therefore, increasing *q*_1_ not only lessens the likelihood of kidnapping, but it also ensures that *q*_1_ stays above *q*_0_ and presents the added benefit of lowering the ransom *D*. This means that increasing *q*_1_ appears to be more optimal than increasing *q*_0_.

A question that might arise is whether it is in the interest of police to advertise the increase in resources, or whether F and K even know about it. From figure 1, K's rational decision (dictated by *e*=1 or *e*=0) to execute or release the hostage, respectively, is known to F. Even if the police remain discreet and do not announce that they are investing more (or less or the same) resources in the case where *e*=1, the events *e*=1 or *e*=0 themselves appear sufficient to result in the bifurcation of *q* into *q*_0_ and *q*_1_. Furthermore, studying this bifurcation allows us to understand better the consequences emanating from increasing either *q*_1_ or *q*_0_. As we have seen earlier, increasing *q*_1_ may result in overall better outcomes.

The non-rational execution of the hostage is a characteristic of Selten's model that can be explained using a Bayesian approach, i.e. by considering the belief K has about F's ability to pay. If K thinks that F can pay but F decides not to, this can result in K reacting in a non-rational way, as proposed by Selten. It is anticipated that by introducing beliefs for K, regarding whether or not F can match his demand, it can lend a further perspective to the analysis of this game. In particular, this would result in considering a Bayesian equilibrium instead of subgame perfect equilibrium.

Selten used a binary decision variable *e* in equation (1.3) in order to describe the situation that K can execute the hostage while enacting a non-rational decision. As the hostage may be executed even when the ransom demand is met, therefore, *α*≠0 even if *C*=*D*. An appropriate probability function to describe the non-rational situation could be when

4.1 $$\alpha =a\left[1-\frac{C}{D}+\beta \right]\;\text{for}\;0\leq C\leq D\;\text{and}\;\beta >0.$$

A policy objective is to minimize the optimal demand as given in equation (3.31). Given fixed resources can be allocated to police to increase the chances of capturing K, these resources are better spent towards increasing *q*_1_.

If K is aware that F cannot meet his demand, then K could either lower his demand and/or decide whether to execute the hostage on rational grounds. This rational decision to execute the hostage depends on probabilities *q*_1_ and *q*_0_, and we know that as long as as *q*_1_>*q*_0_, executing the hostage is not optimal for K. Thus increasing *q*_1_ as opposed to allocating resources to *q*_0_ is again more desirable.

The other situation that could be incorporated in the model is when player K's cost of preparing the kidnapping is considered non-negligible and player F's non-monetary disutilities, other than those incurred by the hostage's life, are however considered negligible. For instance, player F does not attach any value to the capture of the kidnapper.

## Data accessibility

No data were collected in the course of this research.

## References

[1] Rasmusen E 1989 *Games and information, an introduction to game theory*, 3rd edn Cambridge, MA: Basil Blackwell.

[2] Osborne MJ 2003 *An introduction to game theory*. New York, NY: Oxford University Press.

[3] Binmore K 2007 *Game theory: a very short introduction*. New York, NY: Oxford University Press.

[4] Selten R 1976 A simple game model of kidnapping, Working Papers. Institute of Mathematical Economics, vol. 45. Bielefeld, Germany: Center for Mathematical Economics. See https://pub.uni-bielefeld.de/publication/2909646.

[5] Selten R 1973 A simple model of imperfect competition, where 4 are few and 6 are many. *Int. J. Game Theory* 2, 141–201. (doi:10.1007/BF01737566)

[6] Selten R 1965 Spieltheoretische Behandlung eines Oligopolmodells mit Nachfrageträgheit [An oligopoly model with demand inertia]. *Z. die Gesamte Staatswissenschaft* 121, 301–324, 667–689.

[7] Selten R 1975 Reexamination of the perfectness concept for equilibrium points in extensive games. *Int. J. Game Theory* 4, 25–55. (doi:10.1007/BF01766400)

[8] Kalai E, Samet D 1984 Pesistent equilibrium in strategic games. *Int. J. Game Theory* 13, 129–144. (doi:10.1007/BF01769811)

[9] Kuhn HW 1953 Extensive games and the problem of information. In *Contributions to the Theory of Games (AM-28)*, vol. 2 (eds HW Kuhn, AW Tucker) pp. 193–216. Princeton, NJ: Princeton University Press.

[10] Binmore K 2007 *Playing for real: a text on game theory*. Oxford, UK: Oxford University Press.

